# Screening for coeliac disease in children and adults living in a slum of Dhaka, Bangladesh

**DOI:** 10.1136/bmjgast-2019-000294

**Published:** 2019-04-17

**Authors:** Md. Amran Gazi, Subhasish Das, Mustafa Mahfuz, Md. Mehedi Hasan, Md. Shabab Hossain, Shah Mohammad Fahim, Md. Ashraful Alam, Zannatun Noor, Carol A Gilchrist, William A Petri, M Masudur Rahman, Ramendra Nath Mazumder, Rashidul Haque, Shafiqul Alam Sarker, Tahmeed Ahmed

**Affiliations:** 1 Nutrition and Clinical Services Division, icddr,b, Dhaka, Bangladesh; 2 Department of Medicine, Division of Infectious Diseases and International Health, University of Virginia Health System, Charlottesville, Virginia, USA; 3 Department of Gastroenterology, Dhaka Medical College and Hospital, Dhaka, Bangladesh

**Keywords:** coeliac disease, prevalence, bangladesh, ELISA, serology

## Abstract

**Background and objective:**

Serological screening with a confirmation through biopsy has improved the understanding of coeliac disease (CD) epidemiology worldwide. Prevalence of CD in Bangladesh is not yet explored and therefore, we aimed to assess the seroprevalence of CD in slum-dwelling malnourished children and adults in Dhaka.

**Methods:**

Serum samples were collected from three different cohorts: stunted (length-for-age Z-scores (LAZ) <−2) and at risk of stunting children (LAZ <−1 to −2) and malnourished adults (body mass index <18.5 kg/m^2^). Samples from all the participants were assessed for anti-tissue transglutaminase antibody (tTG-IgA) and total serum IgA by ELISA. Positive tTG-IgA and randomly selected low IgA values were reconfirmed using anti-tTG-IgG and gliadin IgG ELISA. CD was diagnosed when second screening tests were found positive and the participants were further investigated by small bowel biopsy.

**Results:**

A total of 818 participants (240 stunted, 272 at risk of stunting children and 306 malnourished adults) were enrolled in the study. Overall, anti-tTG-IgA was positive in 5/818 (0.6%; 95% CI 0.25% to 1.46%). Of the five positive cases, anti-tTG-IgG and gliadin IgG were found positive in only one participant. Duodenal biopsy of positive participant revealed characteristic lesions of CD. Randomly selected low IgA values were found negative in tTG-IgG and gliadin IgG for all the participants. No participant was found total IgA deficient.

**Conclusion:**

The incidence of coeliac autoimmunity is low in malnourished slum dwellers regardless of age in Bangladesh. It is important to investigate the nationwide prevalence to reveal the definite picture.

**Summary box**

**What is already known about this subject?**

- The prevalence of coeliac disease (CD) is known in many countries including India and China, the neighbouring countries of Bangladesh.
- A study conducted on the hospitalised adult patients with irritable bowel syndrome (IBS) in Bangladesh observed a 9% prevalence of CD.
- Two case reports were published on hospitalised patients with CD manifested by chronic diarrhoea, growth failure and chronic liver disease in Bangladesh.

**What are the new findings?**

- This is the first research to report on CD autoimmunity in Bangladeshi children and adults who are malnourished.
- Prevalence of anti-tTG-IgA is 0.6% in slum-dwelling malnourished children and adults in Dhaka, Bangladesh.
- IgA deficiency was not observed among the study participants.

**How might it impact on clinical practice in the foreseeable future?**

- Environmental enteric dysfunction is highly prevalent in children and IBS is common among the general adult population in this region. CD is highly likely to be masked by these syndromes posing a great challenge in defining prevalence, treatment or referral.
- The findings from this study conclude that CD is present in Bangladesh. So, it is important to investigate the nationwide prevalence to reveal the definite picture on the prevalence of CD in the country.

## Introduction

Coeliac disease (CD) is a chronic small intestinal digestive disease caused by an inappropriate immune response to ingested gluten in genetically predisposed people who carry two specific class II human leucocyte antigen (HLA) haplotypes, DQ2/DQ8.^1^ The HLA-DQ2 and HLA-DQ8 present gluten peptide antigens, triggering the activation of T lymphocytes and ultimately leading to mucosal immune responses in intestine.^2^ The classic clinical presentations of CD are mostly gastrointestinal in nature and can be manifested by weight loss, diarrhoea, malnutrition, steatorrhoea and oedema caused by hypoalbuminaemia.^3^ The disease can also be presented with other range of symptoms leading to delayed or missed diagnosis^4–6^ which eventually may result in negative consequences on quality of life.^7 8^

In past reports, the prevalence of CD exhibited evidence of racial and geographical differences, marked by a higher prevalence among the people of European descent.^9^ Geographical variances of CD prevalence can be explained by the differences in HLA-DQ background between the individuals as well as the dietary habit of consuming wheat as staple food. However, the presence of CD-predisposing HLA genes coupled with rapid westernisation of diet (ie, wheat consumption) and the recent global trend of migration might have a contribution to the rise of CD prevalence worldwide.^9^ Bangladesh is also experiencing a nutritional shift, although the staple food is rice along with other food items like wheat, potato, vegetables, fish, meat and milk. The prevalence of CD was observed 1% or a little more in the USA and in several European countries.^10–13^ Data pertaining to prevalence in the Asia-Pacific region are still very limited. A few studies were conducted on the symptomatic individuals in China, India, Malaysia and Vietnam to estimate the prevalence.^14–17^ However, there remains scarcity of information regarding the CD prevalence in Bangladesh especially among the malnourished population. Seroprevalence (IgA tTg positivity) of CD was found 15.38% among severe acute malnourished children in India.^18^ Malnutrition is common in low-income settings like Bangladesh particularly among slum dwellers. CD contributes to the problem of malnutrition because of malabsorption coupled with micronutrient deficiency in these patients.^19^ However, in order to increase awareness among the general population and delineate the real burden of CD to the physicians, the Asian Pacific Association of Gastroenterology and the World Gastroenterology Organization recommended exploring the prevalence of CD across the area.^20^

The gold-standard test to diagnose CD in current clinical practice is to observe characteristic lesion in small intestinal biopsy with a concurrent presence of a CD-specific antibody in blood.^3^ The recent advancement in the diagnostic tools used for CD diagnosis now allows us to estimate the disease prevalence accurately in larger communities by using non-invasive serological markers.^21^ ELISA of IgA class autoantibody against tissue transglutaminase (tTG-IgA) is currently the customary procedure and is a powerful marker for screening of CD.^22^ In order to explore CD prevalence in the Bangladeshi population living in a slum, children aged between 12 and 18 months and adults aged between 18 and 45 years were tested using the tTG-IgA, tTG-IgG, gliadin IgG and total IgA as screening tests.

The primary goal of the current study was to estimate the CD prevalence on the basis of serological assays in young children and adults who are malnourished and residing in a slum of Dhaka, Bangladesh.

## Methods

### Subjects and study population

This study was part of an ongoing Bangladesh Environmental Enteric Dysfunction (BEED) study (ClinicalTrials.gov identifier NCT02812615) which is being conducted among the residents of a slum in Mirpur, one of the 21 regulatory units of Dhaka city. This is a community-based nutrition intervention study, and the details of the overall design of the study have already been published elsewhere.^23^ In short, the goals of this study include the validation of non-invasive biomarkers for assessing gut health, and understanding the pathophysiology and mechanism of environmental enteric dysfunction (EED). In the BEED study, participants were recruited from a cohort of children aged between 12 and 18 months and a cohort of adults aged between 18 and 45 years. The child cohort includes stunted children (length-for-age Z-scores (LAZ) <−2) and at risk of stunting children (LAZ <−1 to −2) and the adult cohort includes malnourished people with body mass index (BMI) <18.5 kg/m^2^. For this particular study, we have taken serum samples from 818 participants collected from July 2016 to March 2018 from the BEED study population. Serum samples had been kept at −80°C in a freezer prior to analysis.

## Study protocol

Serological testing of tTG-IgA was used as primary screening test. If tTG-IgA was found negative, the chance of false-negative cases due to IgA deficiency was considered. Hence, all the participants with negative tTG-IgA were tested for total IgA. In the event with positive tTG-IgA, borderline tTG-IgA, low IgA (randomly selected) or IgA deficiency (IgAD) was found existent, and tTG of isotype IgG (tTG-IgG) and gliadin IgG was assayed for further confirmation of CD. Randomly selected samples from total IgA sufficient group were also assessed for tTG-IgG and gliadin IgG as control (expected to be negative). The CD diagnosis was based on the results of serological tests, histology of small intestine mucosal biopsy and clinical examination of the patient.

### Serum antibody tests

Sera were analysed for tTG-IgA by ELISA using Eu-tTG (Eurospital, Trieste, Italy). All samples were diluted 1:101 in the sample buffer and 6-point calibration curves were used to calculate the levels of antibody, expressed as arbitrary units (units per millilitre). The layout for the interpretation of the results obtained is as follows: positive, ≥16 U/mL; borderline, 9–16 U/mL; normal, <9 U/mL. Quantitative determination of serum IgA was carried out using ELISA, following the manufacturer’s instructions (Affymetrix, eBioscience). Patients with serum levels of <5 mg/dL were considered IgA deficient, whereas values less than the reference limits (<20 mg/dL for children age 1–3 years and <70 mg/dL for adults older than 19 years) were considered as low IgA. Age-related IgA reference intervals are as described by Bienvenu *et al*.^24^ tTG-IgG (Eurospital; ≥20 U/mL positive and <20 U/mL as negative) and gliadin IgG (Inova Diagnostics, San Diego, California, USA; <20 U/mL negative, 20–30 U/mL borderline and ≥30 U/mL as positive) were also analysed by commercial ELISA kit.

## Duodenal biopsy

Subjects found positive in serological tests were informed about the probability of having CD and were requested to consent for performing upper gastrointestinal endoscopy to confirm the diagnosis. Biopsies were taken from the second part of duodenum during endoscopy and fixed in paraffin-embedded 10% buffered formalin. Paraffin sections were then stained by H&E for histopathology. Biopsy samples were graded according to the Marsh classification of 1992.^25^

## Statistical analysis

Statistical analyses were performed using GraphPad Prism. Frequency with proportion was used to summarise categorical variables and mean with SD was used for symmetric quantitative variables. Median and IQR were used for asymmetric numerical variables.

## Results

Overall, 512 children (240 stunted and 272 at risk of stunting) and 306 adults were enrolled into the study. The mean (±SD) age were 14.70±2.09 months for stunted, 14.39±2.08 months for at risk of stunting and 23.68±6.7 years for malnourished adults. The mean (±SD) LAZ for stunted children was −2.78±0.64, for at risk of stunting children was −1.52±0.29 and mean (±SD) BMI for malnourished adults was 17.16±0.87 kg/m^2^. In both stunted and at risk of stunting children, female:male ratio was close to 1:1 whereas 76% were female among malnourished adults. Descriptive features of the study participants are shown in table 1.

**Table 1:** Demographic characteristics of the study population (n=818)

Characteristics	Stunted (n=240)	At risk of stunted (n=272)	Malnourished adults (n=306)
Age (years/months), mean±SD	14.70±2.09	14.39±2.08	23.68±6.7
Female, n (%)	107 (44.77)	149 (54.78)	233 (76.14)
Body mass index (kg/m^2^), mean±SD	NA	NA	17.16±0.87
Weight (kg), mean±SD	7.89±1.83	8.46±0.83	40.91±4.63
Length/height (cm), mean±SD	70.82±2.74	73.52±2.63	154.23±7.67
Length for age, mean±SD	−2.78±0.64	−1.52±0.29	NA
Monthly family income in US$, median (IQR)	144 (120–192)	156 (120–240)	156 (120–216)

**Figure 1:**
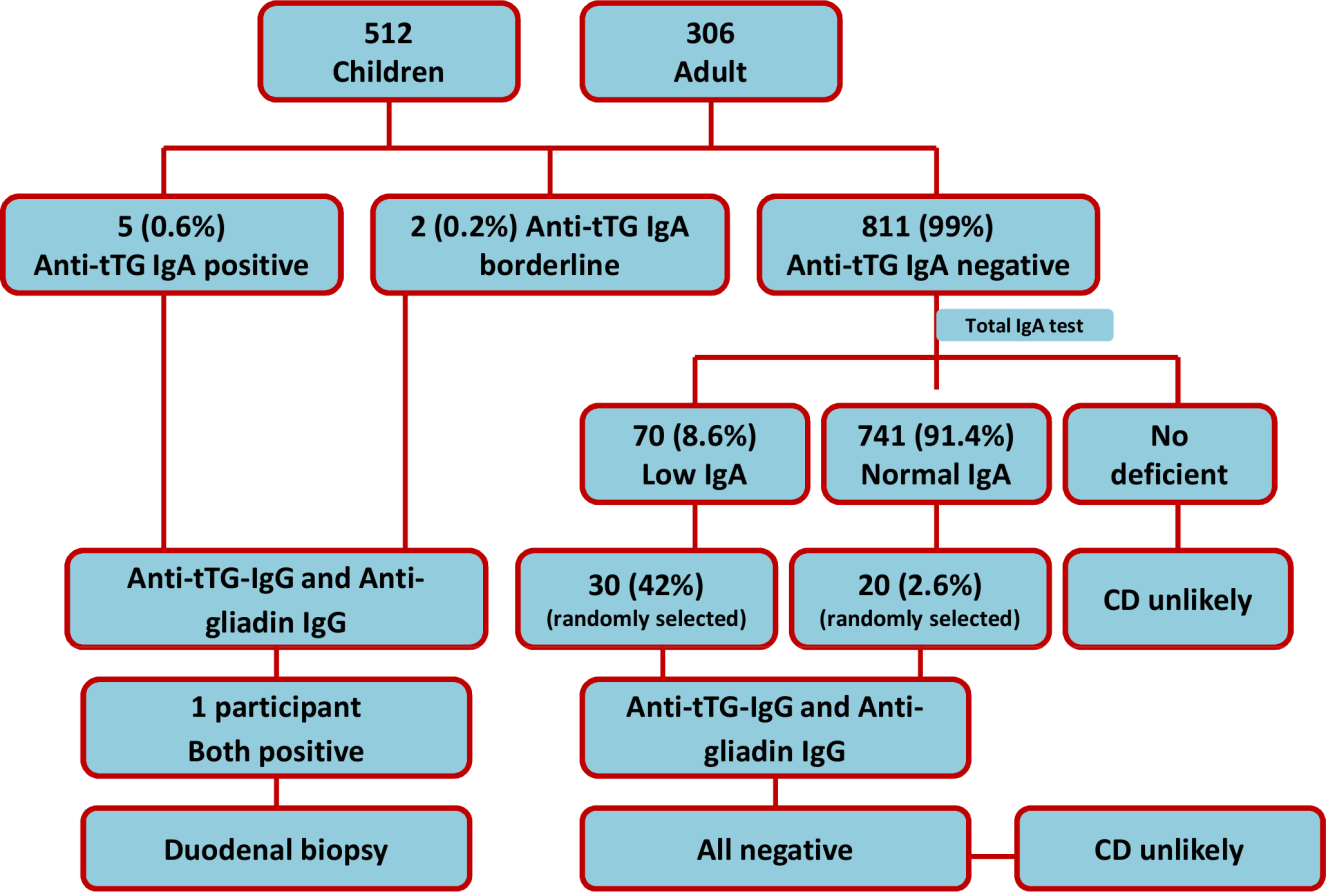
Flow chart showing overall results of the study. CD, coeliac disease.

Anti tTG-IgA was found positive in a total of five participants, two from each stunted and at risk of stunting and one from malnourished adults. Of the five positive cases, three were male and two were female. The values of anti-tTG IgA titre for positive participants were 45.6, 20.8, 17.7, 16.7 and 18.7 U/mL for each of the adult, stunted and children at risk of stunting, respectively. One adult and one stunted participant (both females) had borderline value with antibody titre of 14.2 and 9.3 U/mL, respectively. Most of the participants had normal serum IgA levels. The mean IgA values were 63 mg/dL (range, 7.4–444.4) for the stunted children, 55 mg/dL (range, 7.2–389.2) for at risk of stunting and 208 mg/dL (range, 36.2–504.5) for the adult subjects. However, 70 participants (8.6%) had low IgA while none had IgA deficiency. CD likeliness was further confirmed for five tTG-IgA-positive participants by tTG-IgG and gliadin IgG. However, all were found negative in both tests except one participant from the adult cohort. Furthermore, a random selection of participants who have low and sufficient IgA values was also evaluated for tTG-IgG and gliadin IgG to identify whether there were false-negative cases. However, all were found negative. The overall results are illustrated in figure 1.

The adult male gave consent to undergo endoscopy and his duodenal biopsy revealed characteristic lesions of CD. However, he did not have any remarkable clinical symptoms even on regular consumption of gluten (wheat flour). He was positive for *Campylobacter*-like organism test and was diagnosed to have acute gastritis on endoscopy. The stunted children who were tTG-IgA positive had no significant history of cramps or diarrhoea and their regular diet consisted of the common local dish of *khichuri* (a meal cooked from rice, lentils, vegetables and oil) and breast milk. Both of them refused to give consent for the endoscopic procedure. The remaining two positive participants in tTG-IgA belonged to at risk of stunting cohort. One of them had no significant clinical features whereas the other had history of diarrhoea. These two participants were dropped out from the study. Immunological and clinical findings of the participants who are positive for any serological tests are presented in table 2.

**Table 2:** Clinical profile of the patients positive in IgA anti-tTG

Sex	Cohort	Age	Main clinical findings	Regular diet	tTG-IgA (U/mL)	tTG-IgG (U/mL)	Gliadin IgG (U/mL)	Total IgA (mg/dL)	Endoscopy
M	Stunted child	13 months	Malnutrition, no specific complaints after consuming gluten	Khichuri and breast milk	20.8	13.5	7.8	78	Refused to consent
F	At risk of stunting child	17 months	Malnutrition, no specific complaints after consuming gluten	Khichuri and breast milk	18.7	11.2	6.9	71	Dropped out from study
M	Stunted child	14 months	Malnutrition, no specific complaints after consuming gluten	Khichuri, breast milk, egg, suji, meat, bread, fish, rice	17.7	8.5	2.5	45	Refused to consent
F	At risk of stunting child	13 months	Malnutrition, diarrhoea (1–2 episodes/month), no specific complaints after consuming gluten	Breast milk,*† khichuri, egg, suji, meat, bread, fish, rice	16.7	6.5	3.6	64	Dropped out from study
M	Malnourished adult	20 years	Malnutrition, history of peptic ulcer disease of sibling, no specific complaints after consuming gluten	Rice, bread, lentil, meat	45.6	29.2	67.9	156	Acute gastritis, CLO positive

*Khichuri (hotchpotch) is a local meal cooked from rice, lentils, vegetables and soybean oil.

†*Suji* (semolina) is a traditional dish mixed with milk powder, rice powder, sugar and soybean oil.

CLO, *Campylobacter*-like organism; F, female;M, male.

## Discussion

Our study result demonstrated that CD is present among malnourished children and adults living in a slum of Dhaka, Bangladesh, and the incidence seems to be very low. To our knowledge, this was the first study to explore the prevalence of CD in a Bangladeshi population using specific antibodies.

While the presence of CD is recognised in some countries of Asia, it is still regarded to be uncommon in most of the countries of the same region.^26^ But recent reports have identified the existence of CD in many countries of this region which might be due to the changes in dietary habits. Bangladesh is also rapidly acclimatising to western diet and the actual prevalence of CD in the country is not yet known. Therefore, screening of the disease using sequential sensitive and specific serological tests was carried out both in children and adults. In our study, five participants were found to be anti-tTG-IgA positive, but no one was positive in tTG-IgG and gliadin IgG. IgA deficiency was also not found in these participants.

Previous reports indicated that prevalence of CD has been varied ranging from 1:50 to 1:500 in different countries of the Asia-Pacific region,^27^ including New Zealand, Australia, Syria, Iran, Turkey and Israel. The prevalence was reported extremely low in Japan and China.^28^. CD is well recognised in India, especially in the northern region, where two studies unveiled the incidence to be 0.3% to 1.04%,^29^ although it was believed to be rare in the northeastern and southern parts. A study conducted on adult Indians found seroprevalence of CD in northern (1.23%), northeastern (0.87%) and southern (0.10%) India.^28^ It is known that Eastern India is geographically, socioeconomically, culturally and also in terms of dietary habit close to Bangladesh, strengthening our study findings. According to a systematic review in China,^30^ the number of recorded CD was very low, although a study conducted on diarrhoeal children exhibited histologically proven CD of 12%.^31^ A recent study in China has also shown that approximately 2% of young adults and adolescents (age, 16–25 years) had positive CD serology. However, the population that consume wheat as staple diet exhibited a lower prevalence of CD (0.76%) in Shandong province located in northern China.^15^ Initial reports from Singapore and Vietnam also showed the presence of CD in these countries.^14 17^ A study conducted in Malaysia reported a seroprevalence of 1.25% in healthy young adults.^16^ There has been no report from any representative Bangladeshi sample.^32^ However, one study conducted in hospitalised adults with irritable bowel syndrome demonstrated a 9% prevalence of CD.^33^ The present study employed screening tests to identify CD in individuals living in an urban community and has established the presence of CD among Bangladeshi population.

All the studies that screened CD so far have reported the existence of autoimmunity in variable rates. To circumvent this variation of different autoantibody tests, tTG-IgA test which is the single preferable test for detection of CD had been used as primary test for the assessment of the seroprevalence of this disease. Both the sensitivity and specificity of this test for untreated CD are equal or greater than 95%. Combining several tests to the diagnostic strategy instead of tTG-IgA alone are not recommended in low-risk populations because it might slightly increase the sensitivity but decreases the specificity.^34^ Our study findings depict that the Bangladeshi population falls into the category of low-risk populations. Hence, we have employed tTG-IgG and gliadin IgG as secondary tests only for positive tTG-IgA participants and randomly selected low IgA participants. The lower prevalence reported in our study population perhaps can be explained either by the lower consumption of gluten or the late introduction of gluten in children, and microbiota or exposure to microbes early in childhood that could hinder the immune system development (ie, hygiene hypothesis).^35^ Our study participants were from a slum area in Dhaka, and they live in a poor socioeconomic condition. Monthly median family income for these participants is 1250 taka (equivalent to US$156). So, one may speculate that the lower prevalence is linked to low socioeconomic conditions resulting in, for instance, changes in gut microbiota, the frequency of parasitic and intestinal infections, and various other dietary factors than gluten.

Generally, IgAD is found 10 to 15 times higher in patients with CD (1.30%) compared with the healthy individuals (0.13% to 0.25%), which may yield false-negative results.^17^ In patients diagnosed with low IgA or IgA deficiency, IgG-based testing (IgG DGPs and tTG-IgG) is recommended for further validation,^19^ especially if IgA-based serology assay is negative. In general, total IgAD is defined in most studies as an undetectable serum IgA at a value of 5 mg/dL. This is the lowest measurable concentration fixed by most of the research laboratories.^36^ Partial IgAD is defined as detectable but reduced IgA, higher than 2 SD below normal for age. This is mostly seen in children due to their delayed ontogeny of IgA system after birth. However, this condition is self-limiting and about half of these children reach normal values by 14 years of age (transient IgAD).^37^ We did not apply this definition for partial IgAD in our study. Instead, we only considered low IgA as the value lower than the reference limits by age which in turn helped us to cover more participants in identifying the deficiency. No participants were found to have IgA deficiency; however, 70 participants (8.6%) were found to have low IgA according to the definition. The rest of the participants had normal IgA value. Our results are consistent with the data from other Asian countries such as Japan,^38^ China^39^ and India.^40^ A study conducted in Northern India among adults has detected 6.7% participants with partial IgA deficiency out of 3640 participants screened, which is also in accordance with our findings.^40^ Another study performed in Iran has observed partial IgA deficiency of 7.1% in children (mean age 10.3 years) with rheumatoid arthritis.^41^ We have also found a higher number of participants with low IgA in children. Perhaps, it can be explained by the age of the children enrolled in this study. We have enrolled children less than 2 years of age in this study, and it is established that children aged <2 years are more susceptible to IgA deficiency. However, we have tested tTG-IgG and gliadin IgG to confirm the CD for the participants who had positive tTG-IgA, borderline tTG-IgA value and who had low IgA. Only one participant was positive in both tests which was confirmed by biopsy (CD likely). All others were found negative in tTG-IgG and gliadin IgG and hence CD was unlikely for them.

The other test that can be considered for CD is HLA typing as the HLA-DQ2 heterodimer encoded by the DQA1*05 and DQB1*02 alleles is frequent in patients with CD. However, according to the Allele Frequency Net Database (www.allelefrequencies.net), this should not be common in the Bangladeshi population and neither is the HLA-DQ8 heterodimer which comprises the second most common HLA type.^42^ The sample set entered into the database was not extensive and therefore we did not consider this test in our study. The low specificity of this test has also been borne in mind.^43^

In conclusion, the prevalence of CD was found to be low among children and adults in the present study. However, the study population belongs to the lower socioeconomic group and their diet might be different from the average Bangladeshi diet. Moreover, gastrointestinal infection and infestations are common among the study population. Western diet is getting more popular particularly among the middle-class and affluent people. Therefore, the prevalence may be different among the general population. Our study findings hence emphasised the necessity of nationwide investigation of the CD prevalence both in children and adults of the general population and the population who belongs to poor socioeconomic status and living in inimical environment because CD symptoms in this group can easily be obscured by the commonly observed environmental enteropathy, characterised by persistent diarrhoea, failure to thrive and malnutrition.^44^

## Limitations

There were some limitations in this study. First, we could not collect the biopsy specimen from all the participants who were serology positive. This was due to refusal of the participants to undergo endoscopy and attrition of the participants during the study. Second, the target population was stunted or at risk of stunting children and malnourished adults and therefore by definition had not been completely healthy. Another drawback of the study was that the secondary screening tests (tTG-IgG and gliadin IgG) were not employed for all the participants in this study. Cut-off values selected for CD serum antibody assays were another limitation of our study as that had been chosen according to the recommendations of the manufacturers and might be different in this population. Lastly, the lower number of samples was another limitation because the study was conducted on the population of a certain area.

## Data Availability

All data relevant to the study are included in the article or uploaded as online supplementary information.
